# Supplementary material for: Whole-Genome Sequencing Analysis of a stx-Negative Escherichia coli O63:H6 Isolate Associated with Hemolytic Uremic Syndrome
Source: Diagnostics (Basel). 2021 Oct 2;11(10):1823. doi: 10.3390/diagnostics11101823 (PMC8534868; doi:10.3390/diagnostics11101823)
Supplement: Supplementary file 1 [file diagnostics-11-01823-s001.zip › diagnostics-1365473-supplementary.pdf]

Supplementary Materials

Supplementary Table S1. Primers used for detection of *stx1*, *stx2*, *stx2f*, and *eae*

Target gene	Sequence (5`→3`)
stx1	CGTACGGGGATGCAGATAAATCGC
	CAGTCATTACATAAGAACGCCCAC
stx2	GTTCTGCGTTTTGTCACTGTCAC
	GTCGCCAGTTATCTGACATTCTGG
stx2f	AGATTGGGCGTCATTCACTGGTTG
	TACTTTAATGGCCGCCCTGTCTCC
eae	CCCGAATTCGGCACAAGCATAAGC
	CCCGGATCCGTCTCGCCAGTATTCG
